# Cascading effects of climate change on plankton community structure

**DOI:** 10.1002/ece3.6055

**Published:** 2020-02-05

**Authors:** Grace E. P. Murphy, Tamara N. Romanuk, Boris Worm

**Affiliations:** ^1^ Department of Biology Dalhousie University Halifax NS Canada

**Keywords:** acidification, global change, indirect effects, plankton, warming

## Abstract

Plankton communities account for at least half of global primary production and play a key role in the global carbon cycle. Warming and acidification may alter the interaction chains in these communities from the bottom and top of the food web. Yet, the relative importance of these potentially complex interactions has not yet been quantified. Here, we examine the isolated and combined effects of warming, acidification, and reductions in phytoplankton and predator abundances in a series of factorial experiments. We find that warming directly impacts the top of the food web, but that the intermediate trophic groups are more strongly influenced by indirect effects mediated by altered top‐down interactions. Direct manipulations of predator and phytoplankton abundance reveal similar strong top‐down interactions following top predator decline. A meta‐analysis of published experiments further supports the conclusion that warming has stronger direct impacts on the top and bottom of the food web rather than the intermediate trophic groups, with important differences between freshwater and marine plankton communities. Our results reveal that the trophic effect of warming cascading down from the top of the plankton food web is a powerful agent of global change.

## Graphical abstract

Plankton communities account for at least half of global primary production and play a key role in the global carbon cycle. Warming and acidification may alter the interaction chains in these communities from the bottom and top of the food web. Here, we examine the isolated and combined effects of warming, acidification, and reductions in phytoplankton and predator abundances in a series of factorial experiments. We find that warming directly impacts the top of the food web, but that the intermediate trophic groups are more strongly influenced by indirect effects mediated by altered top‐down interactions.

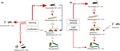

## 1 INTRODUCTION

Plankton communities play a crucial global role by contributing at least half of global primary production, forming the foundation of aquatic food webs, transferring biomass to higher trophic levels, and influencing the global carbon cycle (Chassot et al., 2010; Falkowski, Barber, & Smetacek, 1998; Roemmich & McGowan, 1995). Several studies have demonstrated significant impacts of climate change on plankton communities, including decreased productivity (Boyce, Lewis, & Worm, 2010) and a shift from autotrophic to heterotrophic dominance (O'Connor, Piehler, Leech, Anton, & Bruno, 2009; Sommer & Lewandowska, 2011). Evidence of how climate change, most notably elevated temperature and decreased pH, impact planktonic organisms is growing. Direct effects of warming and acidification to plankton include changes in physiology and behavior that can alter growth (McFeeters & Frost, 2011), body size (Garzke, Ismar, & Sommer, 2015; Sommer, Peter, Genitsaris, & Moustaka‐Gouni, 2016), reproduction (Weydmann, Søreide, Kwasniewski, & Widdicombe, 2012), and survival (Cripps, Lindeque, & Flynn, 2015; Kroeker, Kordas, Crim, & Singh, 2010).

In addition to directly affecting planktonic organisms, warming and acidification can alter the structure of plankton communities by impacting the trophic interaction chains of plankton‐based food webs (Gaedke et al., 2010). More generally, we know that complex interaction chains are arising from human‐driven environmental change, which include both direct and indirect effects (Gilman, Urban, Tewksbury, Gilchrist, & Holt, 2010; Worm & Paine, 2016). Direct responses to environmental change can propagate along trophic interaction pathways and result in cascading effects (Nagelkerken, Goldenberg, Ferreira, Russell, & Connell, 2017). Plankton communities can be influenced by either strengthened top‐down or bottom‐up control following climate change drivers (Kratina, Greig, Thompson, Carvalho‐Pereira, & Shurin, 2012). For example, top‐down control can intensify in communities where climate change causes food demand increases in plankton consumers (O'Connor et al., 2009; Sommer & Lewandowska, 2011), while altered primary production can intensify bottom‐up control of plankton communities (Gruner et al., 2008).

Few climate change studies have addressed the potential for indirect trophic interactions to shape the structure of multitrophic plankton communities. Trophic interactions can considerably impact community responses to climate change and are thought to be as important, or potentially more important, as direct nontrophic effects in shaping ecological communities (Alsterberg, Eklof, Gamfeldt, Havenhand, & Sundback, 2013; Wernberg, Smale, & Thomsen, 2012). For example, Suttle, Thomsen, and Power (2007) showed that species interactions more strongly influenced grassland community responses to changing precipitation compared to the direct effects of altered precipitation regimes. Specifically, for plankton communities, Gaedke et al. (2010) showed that indirect biotic interactions played a stronger role than direct climate effects in structuring a phytoplankton community following experimental warming. Studies that consider both the direct and indirect effects of environmental change are needed to understand the complex ways in which climate change is likely to alter plankton communities.

Here, we use two parallel factorial experiments of global change drivers to disentangle the single and combined impacts of global change on plankton community structure. In Experiment A, we combine warming with acidification, and in Experiment B, we combine predator removal to simulate top predator loss with phytoplankton biomass decline to similar changes in primary production. We then use path analysis to investigate causal pathways between global change drivers and trophic group densities. Specifically, we examine the strength of direct pathways between drivers and trophic groups and compare these to the pathway strengths between different trophic groups, to test whether indirect biotic interactions can mediate the direct effects of drivers on trophic group densities. We also use path analysis to test whether top‐down or bottom‐up control play a stronger role in structuring the plankton community. Finally, we employ a meta‐analysis of experimental warming studies to explore the generality of our results across widely different plankton communities, both freshwater and marine.

Path analysis is a subset of structural equation modeling (SEM) in which only directly measured variables are included in the model. SEM is a powerful multivariate tool that has many applications in ecological research as it allows for the evaluation of causal hypotheses using empirically derived data (Grace, 2006). In SEM, a priori models are constructed as a series of cause–effect pathways connecting measured variables. Paths are determined based on which predictor variables are assumed a priori to affect other variables. From this, the covariance matrix derived from empirical data is used to test the strength of pathways in the model. SEM is unique from other types of multiple regression as it allows a variable to be both influenced by and influence other variables, thus creating a network of interactions (Grace, 2006). Also, SEM is a uniquely suited technique for exploring the effects of global change drivers on community structure as it partitions direct and indirect effects. Therefore, we can gain insight into the extent that indirect trophic effects mediate the net effects of global change drivers on the plankton community by comparing the net effects obtained from ANOVAs with the partitioned effects estimated from path analyses (Alsterberg et al., 2013; Antiquiera, Petchey, & Romero, 2018). Here, we use this tool to detect the relative influence of direct and indirect effects in shaping the response of plankton communities to climate warming and acidification.

## 2 METHODS

### 2.1 Study system

To establish a natural community, we collected rock pool plankton communities from Prospect, Nova Scotia, Canada (43°29′26″N, 65°43′10″W), in October 2012. Rock pools are common in the supralittoral zone along rocky coasts around the world. Laboratory analogues of natural rock pool communities are well‐established experimental models that have been extensively used to address multitrophic responses to species loss (Campbell, 2010; Coll & Hargadon, 2012), warming (Tuck & Romanuk, 2012), species invasions (Romanuk & Kolasa, 2005), nutrient enrichment (Romanuk, Vogt, & Kolasa, 2006), and other environmental changes. Small size, ease of manipulation, contained structure, and short generation time of organisms make rock pool microcosms ideal model ecosystems for exploring the impacts of global change on plankton community structure (Srivastava et al., 2004). The communities inhabiting the microcosms in our experiments were constructed from natural rock pool assemblages populated by algae, phytoplankton, zooplankton, and small invertebrates.

### 2.2 Experimental design

To investigate the trophic interactions between the plankton groups, we first classified ten zooplankton species into functional groups based on five trophic roles (Figure S1). We determined feeding interactions between species from previous feeding trials (Tuck & Romanuk, 2012) and literature review. Two predators occupy the community, a cyclopoid copepod (*Microcyclops varicans*), which is the top predator, and a predatory flatworm (*Gyratrix* sp.), which occupies the role of mesopredator. Herbivores are represented by cladocerans (*Alona *sp.), nematodes, and an amphipod (*Gammarus oceanicus*). Omnivores are represented by calanoid copepods (*Acartia *sp.), and an ostracod (*Cyprinidae eucypris*). Detritivores are represented by harpacticoid copepods, an oligochaete (*Limnodrilus hoffmesteri*), and an aquatic springtail (*Archisotoma *sp.).

Once collected, communities were held in culturing aquaria for three weeks and then transferred to 90 1,500‐ml microcosms. Microcosms were maintained at 21°C on a full‐spectrum 12‐hr light/dark cycle. After a two‐week acclimation period, the zooplankton communities in several of the microcosms had diverged from the composition present in the majority of the microcosms. Therefore, we chose 72 microcosms that had similar zooplankton community compositions to use in the experiments, resulting in four replicates for each treatment. Previous studies using similar rock pool communities have used four to six replicates per treatment (Campbell, 2010; Coll & Hargadon, 2012, Tuck & Romanuk, 2012) so we expect that four replicates are sufficient to capture variability. Water volume in the microcosms was kept constant throughout the experiments by adding filtered rock‐pool water, when necessary.

We conducted two parallel experiments that each followed a 3 × 3 factorial fully crossed design. In Experiment A, we elevated the microcosm water temperature by 0, 4, or 8°C and reduced the pH by 0, 0.4, or 0.8 pH units. Mean surface temperature is predicted to increase by 2–5°C over the next century (IPCC, 2013). Therefore, we chose levels of experimental warming to represent a predicted warming scenario (4°C) and an extreme warming scenario (8°C). This range of temperature increase has been used in other shallow aquatic ecosystem warming experiments (Sampaio, Rodil, Vaz‐Pinto, Fernandez, & Arenas, 2017; Tuck, 2010; Yvon‐durocher, Montoya, Trimmer, & Woodward, 2010). Mean ocean pH is expected to drop by 0.14–0.35 units from increased CO_2_ concentrations over the next century (IPCC, 2013). We note that rather than manipulating acidification using CO_2_ addition, we use the addition of sulfuric acid to reduce pH. Thus, this treatment is not simulating the effects of ocean acidification but instead simulating the effects of pH decline from issues like acid precipitation and pollution run‐off. Acid precipitation and pollution runoff can result in larger pH declines to aquatic ecosystems than CO_2_ deposition alone, with a 2–3 unit drop possible in extreme scenarios (Donahue, Schindler, Page, & Stainton, 1998; Singh & Agrawal, 2008). Therefore, the levels of experimental pH decline we chose are in line with that predicted to occur from increased CO_2_ concentrations, however, are on the lower range of what can occur from acid precipitation and pollution runoff.

In Experiment B, we directly manipulated top predator abundance and phytoplankton density to simulate the negative impact that warming is expected to cause on the top and bottom of the food web. The rationale for Experiment B was to analyze the effects of the biotic changes that typically occur with warming, while eliminating the abiotic influences of warming on the plankton community. Experiment B consisted of three predator removal treatments: no removal, 50% removal, and 100% removal, crossed with three light reduction treatments: 12 hr of light per day, 6 hr of light per day, and 3 hr of light per day. Further description of experimental manipulations is provided in Appendix S1. Both experiments ran for 8 weeks. Given the short generation times of the zooplankton species, the 8‐week period allowed the study of community dynamics over several generations.

We monitored zooplankton density by performing weekly live counts on two 50‐ml subsamples from each microcosm. After counting the individuals present in the subsamples, the entire contents of the microcosm were observed under a stereomicroscope to determine whether a species was present in the microcosm but absent in the subsample. If this was the case, then the species density was recorded as 0.5 to represent its presence at low abundance, in the community. Similar sampling methods have been used to monitor zooplankton density in other studies utilizing these same experimental systems (Campbell, 2010; Tuck, 2010; Tuck & Romanuk, 2012). Total chlorophyll‐a concentration and the concentration of four algae classes (green algae, diatoms, cryptophyta, and cyanobacteria) was measured weekly from two 25‐ml subsamples using a laboratory spectrofluorometer (bbe‐moldaenke).

### 2.3 Statistical analyses

Trophic group densities were averaged across the duration of the experiment, excluding the pretreatment counts (Figures S4 and S5). We conducted two‐way multivariate permutation‐based analysis of variance (PERMANOVA) to test the single and interactive effects of (a) warming and acidification and (b) predator removal and light reduction on the trophic composition of the zooplankton community. Trophic group density data were log_10_ (*x* + 1)‐transformed to scale down densities of highly abundant trophic groups and increase the importance of less dense trophic groups in the analysis. The PERMANOVA was conducted on a Bray–Curtis similarity matrix. We used a zero‐adjusted Bray–Curtis similarity matrix for the trophic group density data to dampen the fluctuations of the metric from near‐blank samples (Clarke & Gorley, 2006). Residuals were permutated under a reduced model with 999 permutations. The null hypothesis was rejected when *p* < .05.

We conducted factorial two‐way ANOVAs to analyze the single and interactive effects of (a) warming and acidification and (b) predator removal and light reduction on the zooplankton and phytoplankton trophic group densities. All data were log_10_ (*x* + 1)‐transformed prior to analyses to assure homogeneity of variance and normality. The null hypothesis was rejected when *p* < .05.

We used path analyses to disentangle the causal relationships between variables and quantify the indirect and direct effects of warming, acidification, predator removal, and light reduction on the trophic composition of the experimental plankton communities. Path analysis uses the covariance matrix derived from variables measured in the experiments (i.e., temperature, pH, and trophic group densities) to partition relationships between variables based on a priori hypothesized interaction pathways (Grace, 2006).

For each experiment, we developed two a priori models to describe the hypothesized causal mechanisms for how the experimental treatments, and subsequent biotic interactions, interact to alter plankton trophic composition. The first model included top‐down controls on the plankton community and the second model included bottom‐up controls on the plankton community. The full a priori models constructed for Experiment A contained paths from warming and acidification variables to all trophic group density variables as we assumed all plankton trophic groups may be affected by warming and acidification (Figure S2). The full a priori models constructed for Experiment B contained paths from predator removal only to the top predator trophic group and light reduction only to the phytoplankton trophic group (Figure S3). Pathways between trophic groups were the same for both experiments and were based on known feeding interactions determined through a combination of previous feeding trials (Tuck & Romanuk, 2012) and literature reviews (Figure S1).

In both experiments, we compared two alternative models: top‐down control and bottom‐up control between consumers and prey. In each scenario, we constructed a full model with all potential pathways and subsequently removed pathways without strong support, that is, those that were not significantly different from zero (*p* > .5). We compared top‐down and bottom‐up models using the sample size‐corrected Akaike information criterion (AICc). The AICc considers model complexity compared to improvement in model fit to determine the most parsimonious model, with lower AICc values indicating better fit (Claeskens & Hjort, 2008). We use a ∆AICc > 2 to determine whether there is sufficient support for the model with the lower AICc having better fit. We also assess model fit using Shipley's test of d‐seperation and Fisher's *C* statistic. We report standardized path coefficients, which indicate the relative strength and direction of the direct relationship between variables. Indirect effects are the product of path coefficients through all mediator variables, and total effects are the sum of direct and indirect pathways. Model parameters were estimated using maximum likelihood.

PERMANOVAs were conducted in PRIMER (version 7.1) with PERMANOVA+ (version 1.0, Clarke & Warwick, 2001). ANOVAs were conducted in R version 3.2.2 (R Development Core Team, 2015). SEM analyses were performed using the *piecewiseSEM* package in R (Lefcheck, 2016).#

### 2.4 Meta‐analysis

We used a meta‐analysis of published warming experiments to determine whether our results are generalizable across other marine and freshwater plankton climate change studies. We focused our meta‐analysis on warming studies as there were not sufficient acidification, predator removal, or light reduction studies that fit our inclusion criteria in the published literature. We searched for studies that measured the effect of warming on the density or biomass of predatory zooplankton, herbivorous zooplankton, and phytoplankton. We performed a literature search using the ISI Web of Science database of the following research areas: “environmental sciences,” “ecology,” “biodiversity conservation,” “marine freshwater biology,” and “oceanography.” We used the following search expressions: “warming” OR “climate change” OR “temperature increase” AND (“plankton” OR “trophic cascade”). We also searched the references of relevant publications. A final search of the literature was completed in June 2016.

To be included in our analysis, studies had to provide density or biomass measurements for all three trophic groups in both an ambient and experimentally warmed treatment. When measures were reported for multiple sampling dates, we averaged values across dates. We evaluated the impact of warming on the trophic group density/biomass across studies in two ways. First, we performed a weighted random effects meta‐analysis using the commonly used log response ratio $\left[\text{LRR}\;=\;\text{ln}\left({\bar{X}}_{\text{warmed}}/{\bar{X}}_{\text{ambient}}\right)\right]$ as the effect size, to compare the net effects of warming on the densities of the three trophic groups. A total of 16 studies representing 28 responses to warming for each trophic group were included (Table S3). We used boxplots to assess outliers and removed points that were outside 1.5 times the interquartile range. Effect sizes were weighted according to their sampling variances, and the *Q*‐statistic was used to test for effect size heterogeneity.

Second, we quantified the direct and indirect effects of warming on the trophic groups using path analysis to fit the data from each individual study to an a priori model. The standardized path coefficients obtained from each path analysis were used as effect sizes in a weighted random effects meta‐analysis. The use of standardized path coefficients from SEMs as effect size estimates in meta‐analyses has recently been utilized in other ecological studies to evaluate the generality of direct and indirect effects (Garcia‐Palacios et al., 2015; Lewandowska et al., 2016). This technique requires the use of full datasets as opposed to summary statistics that can be used in net effect meta‐analyses. We assembled 12 full datasets from the 28 responses included in the net effect meta‐analysis described above to use in the SEM analysis. The a priori model was evaluated separately for each study, and we used chi‐square tests to determine how well each dataset fit. Only models that did not significantly differ from the a priori model were included in the meta‐analysis (*n* = 10). The results including all models (*n* = 12) are shown in Figure S4. Effect sizes were weighted according to their sampling variances, and the *Q*‐statistic was used to test for effect size heterogeneity. Models were fit in the *piecewiseSEM* package in R (Lefcheck, 2016). Meta‐analyses were conducted in the *metafor* package in R (Viechtbauer, 2010).

## 3 RESULTS

### 3.1 Experiment A: warming × acidification

Multivariate PERMANOVA detected a significant effect of warming and acidification on trophic group composition of the zooplankton community (warming pseudo‐*F* = 54.86, *p* = .001; acidification pseudo‐*F* = 5.08, *p* = .002). A significant interaction between warming and acidification on the trophic composition of the zooplankton community was also detected (pseudo‐*F* = 3.23, *p* = .004).

**Figure 1:**
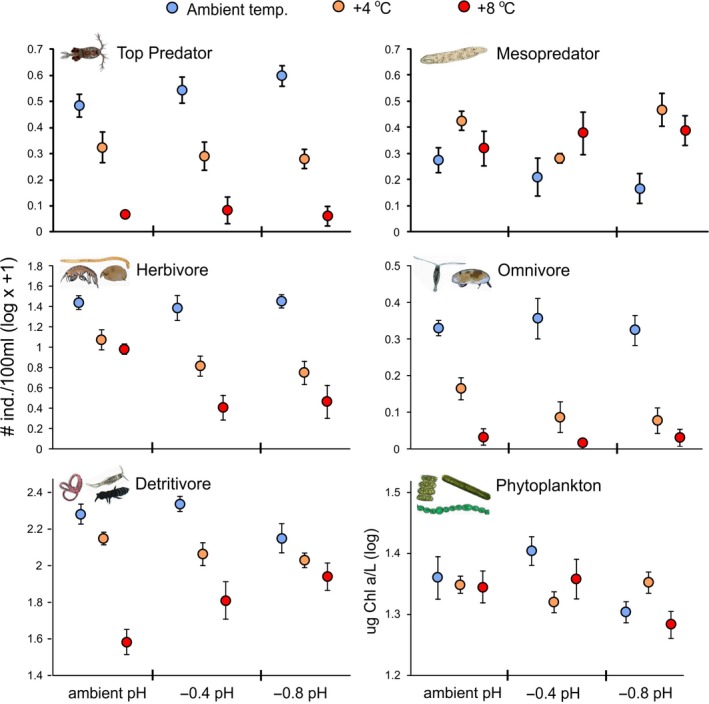
Response of different trophic groups to warming and acidification (Experiment A). Average densities of six trophic groups (mean ± 1*SE*, *n* = 4) are shown for each experimental treatment

ANOVA indicated that warming significantly reduced the density of all zooplankton trophic groups (*p* < .001) with the exception of the mesopredator flatworms, which had significantly higher density in the warmed treatments (*p* = .001; Figure 1; Table 1). Herbivores were the only zooplankton trophic group impacted by acidification alone, with significantly lower herbivore density in the acidification treatments (*p* = .002). We observed an interaction between warming and acidification on detritivore density (*p* = .005) and a marginally insignificant interaction on herbivore density (*p* = .086); yet the direction of the effect was opposite for the two groups. Acidification strengthened the negative effect of warming on herbivore density, while acidification dampened the negative effect of warming on detritivore density (Figure 1).

**Table 1:** Results of two‐way ANOVA testing the effects of warming, acidification, and their interaction on the average densities of zooplankton and phytoplankton groups

	Warming		Acidification		Warming × Acidification	
	*F*	*p*	*F*	*p*	*F*	*p*
Top predator	110.76	**<.001**	0.413	.745	0.927	.493
Mesopredator	9.15	**<.001**	0.685	.57	1.64	.179
Herbivore	63.97	**<.001**	7.03	**.002**	2.14	.086
Omnivore	80.72	**<.001**	0.945	.435	0.781	.593
Detritivore	50.05	**<.001**	0.761	.527	4.2	**.005**
Total zooplankton	61.32	**<.001**	0.226	.877	3.24	**.018**
Green algae	4.83	**.017**	9.03	**<.001**	5.29	**<.001**
Cyanobacteria	6.29	**.006**	1.05	.39	1.77	.147
Diatom	35.99	**<.001**	6.36	**.003**	5.75	**<.001**
Cryptophyta	4.82	**.017**	5.94	**.028**	0.635	.7
Total phytoplankton	1.08	.357	3.08	**.047**	2.018	.103

Note

All densities were log (*x* + 1)‐transformed for the analyses. Significant effects (*p* < .05) are in bold.

Acidification, but not warming, nor the combination of acidification and warming, influenced total phytoplankton concentration, although we only observed a significant decline in total phytoplankton in the severe acidification treatment (*p* = .049), and not the moderate acidification treatment (*p* = .746). Warming and acidification had varying effects of the concentration of the four classes of phytoplankton (Table 1). The absence of a significant effect of warming on total phytoplankton concentration was due to the countervailing effect of warming on the different phytoplankton classes. Warming significantly increased the concentration of cryptophyta (*p* = .017) and diatoms (*p* < .001), but significantly decreased the concentration of green algae (*p* = .017) and cyanobacteria (*p* = .006).

### 3.2 Experiment B: top predator removal × light reduction

Multivariate PERMANOVA detected a significant effect of top predator removal and light reduction, but no interaction between the factors, on the trophic composition of the zooplankton community (removal pseudo‐*F* = 6.92, *p* = .001; light reduction pseudo‐*F* = 2.76, *p* = .018).

**Figure 2:**
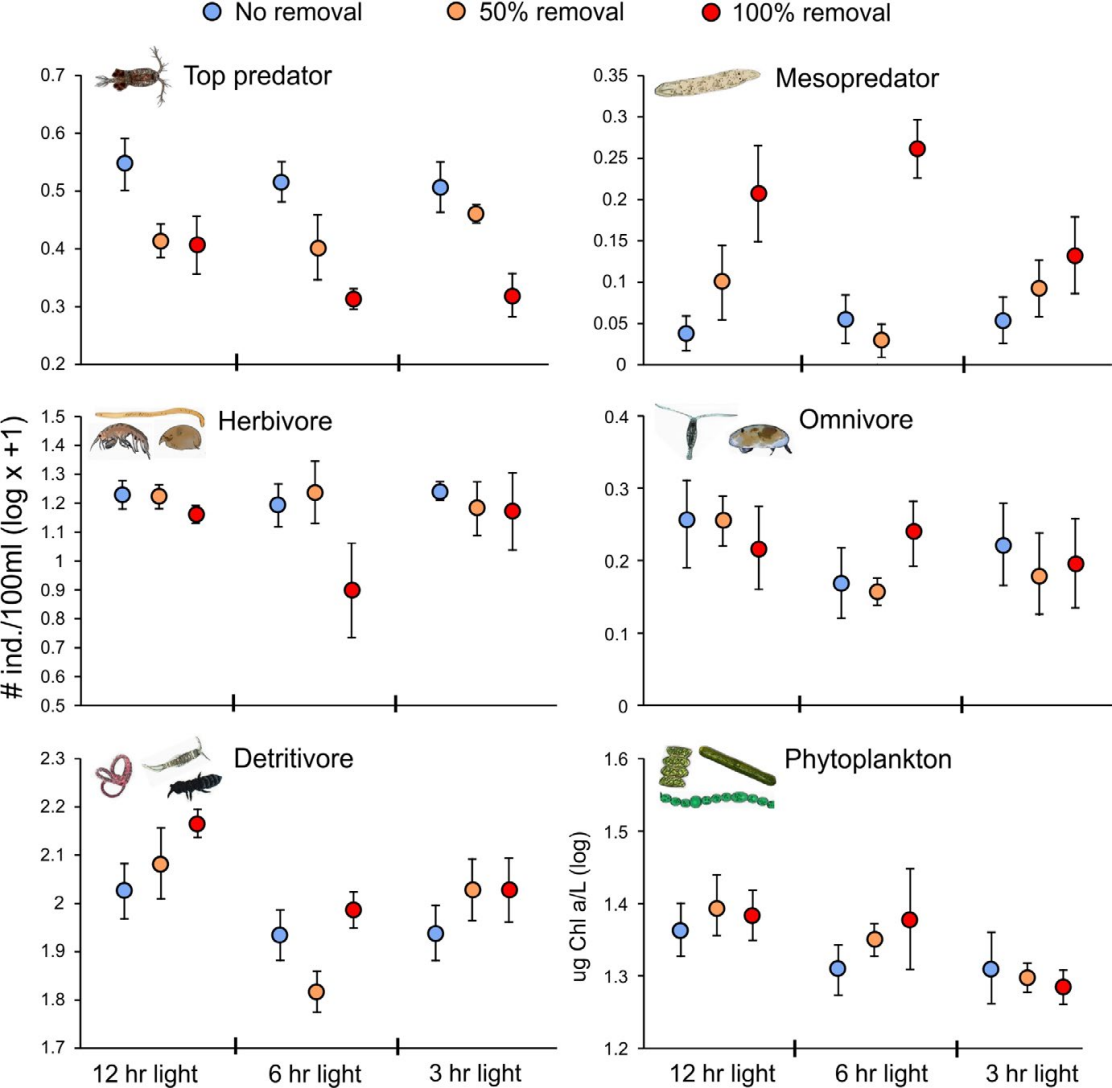
Response of different trophic groups to top predator removal and light reduction (Experiment B). Average densities of trophic groups (mean ± 1*SE*, *n* = 4) are shown for each experimental treatment

ANOVA indicated that the density of the mesopredator flatworm was significantly higher in the 100% predator removal treatment (*p* < .001), but not in the 50% predator removal treatment, compared to no removals (Figure 2). Top predator removal also resulted in a marginally insignificant decline in herbivore density (*p* = .087) and a marginally insignificant increase in detritivore density (*p* = .06), while no effect of removal was observed for the abundance of omnivore zooplankton (Table 2). We observed no significant effects of the predator removal treatments on total phytoplankton density or the density of any of the phytoplankton taxa (Table 2).

**Table 2:** Results of two‐way ANOVA testing the effects of predator removal, light reduction, and their interaction on the average densities of zooplankton and phytoplankton groups

	Removal		Light		Removal × Light	
	*F*	*p*	*F*	*p*	*F*	*p*
Top predator	19.32	**<.001**	1.23	.308	1.04	.404
Mesopredator	15.89	**<.001**	0.403	.673	2.17	.099
Herbivore	2.68	.087	1.14	.334	1.15	.356
Omnivore	0.118	.889	0.921	.41	0.53	.717
Detritivore	3.13	.06	9.35	**<.001**	1.39	.264
Total zooplankton	1.88	.172	9.37	**<.001**	1.03	.409
Green algae	0.536	.591	4.317	**.024**	0.321	.861
Cyanobacteria	0.346	.711	18.7	**<.001**	1.68	.184
Diatom	0.818	.452	0.726	.493	0.296	.878
Cryptophyta	0.964	.394	23.17	**<.001**	1.94	.133
Total phytoplankton	0.29	.751	3.78	**.036**	0.485	.746

Note

All densities were log (*x* + 1)‐transformed for the analyses. Significant effects (*p* < .05) are in bold.

The only zooplankton trophic group significantly impacted by light reduction was the detritivores, where we observed significantly lower density in the 6‐hr light treatment compared to the control (*p* < .001). We found no interactive effects of predator removal and light reduction on either the zooplankton or phytoplankton trophic groups (Table 2).

### 3.3 Structural equation models

#### 3.3.1 Experiment A: warming × acidification

**Figure 3:**
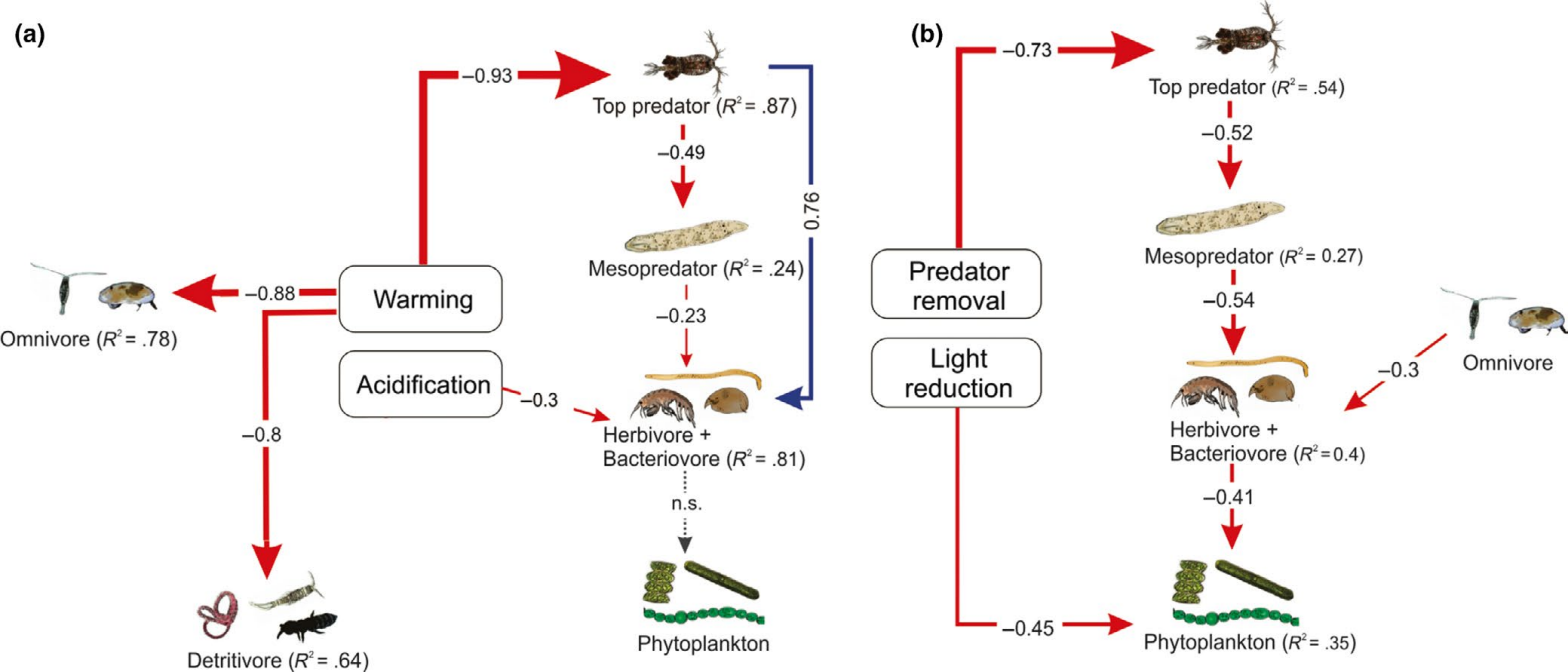
Path diagrams showing how (a) warming and acidification and (b) top predator removal and light reduction are associated with changing consumer and producer densities. Results are from a fitted structural equation model. Standardized path coefficients are shown next to each path and indicate the relative strength of the relationship between variables. Solid paths are statistically different from 0 at *p* < .05, and line thickness is proportional to the relative weight of the standardized path coefficient. Red lines represent significant negative relationships, blue lines represent significant positive relationships, and dotted lines represent nonsignificant effects. Percentages represent the explained variance. All densities were log(*x* + 1)‐transformed prior to analysis. Temperature and pH were square root‐transformed prior to analysis. Model selection steps shown in Tables S4–S7. Final model summary statistics shown in Tables S8 and S9

In Experiment A, the top‐down control model provided the best fit to the data after stepwise removal of nonsignificant pathways (AICc_top‐down_ = 125.92, AICc_bottom‐up_ = 131.5). Structural equation models indicated strong direct effects of warming on top predators (direct path coefficient warming → top predator = −0.93), omnivores (direct path coefficient warming → omnivore = −0.88), and detritivores (direct path coefficient warming → detritivore = −0.8 but no direct impacts of warming on mesopredators, herbivores, or the phytoplankton trophic group; Table S1, Figure 3). Instead, the SEM suggests that the significant net increase in mesopredator density from warming that was reported in the ANOVA was indirectly driven by reduced top predator density (indirect path coefficient warming → mesopredator = +0.46; Table S1). The SEM also suggests that the net decline in herbivore density from warming that was reported in the ANOVA was primarily driven by indirect effects of cascading trophic interactions. These negative indirect effects originated from a decline in top predator density, which negatively affected herbivore density through both an increase in mesopredator density (indirect path coefficient warming → herbivore = −0.10; Table S1), and a reduction in the direct positive interaction between top predators and herbivores (indirect path coefficient warming → herbivore = −0.71; Table S1). These two indirect effects from warming to herbivore density resulted in a relatively large overall indirect effect of warming on herbivore density (overall indirect path coefficient warming → herbivore = −0.81; Table S1). The indirect effect of warming on mesopredator and herbivore densities revealed in the SEM, and the stronger support of a top‐down compared to bottom‐up model, suggests that warming resulted in a top‐down trophic cascade (Figure 3). The trophic cascade did not extend to phytoplankton, with a nonsignificant pathway between herbivore and phytoplankton density.

#### 3.3.2 Experiment B: top predator removal × light reduction

In Experiment B, the top‐down control model also provided the best fit to the data after stepwise removal of nonsignificant pathways (AIC_top‐down_ = 83.91, AIC_bottom‐up_ = 89.06). The strong direct effects of predator removal on top predator density (direct path coefficient predator removal → top predator = −0.73; Table S2) and light reduction on phytoplankton density (direct path coefficient light reduction → phytoplankton = −0.45; Table S2) are intuitive given that the treatments were designed to alter the densities of these trophic groups. Instead, we used the SEM in this experiment to evaluate interaction strengths in the intermediate trophic groups. Similar to the indirect effects of warming on intermediate trophic groups revealed by the SEM for Experiment A, the SEM for Experiment B revealed an indirect positive effect of top predator decline on mesopredator density (indirect path coefficient removal → mesopredator = +0.38; Table S2), and an indirect negative effect on herbivore density (indirect path coefficient removal → herbivore = −0.20; Table S2, Figure 3b).

### 3.4 Meta‐analysis

**Figure 4:**
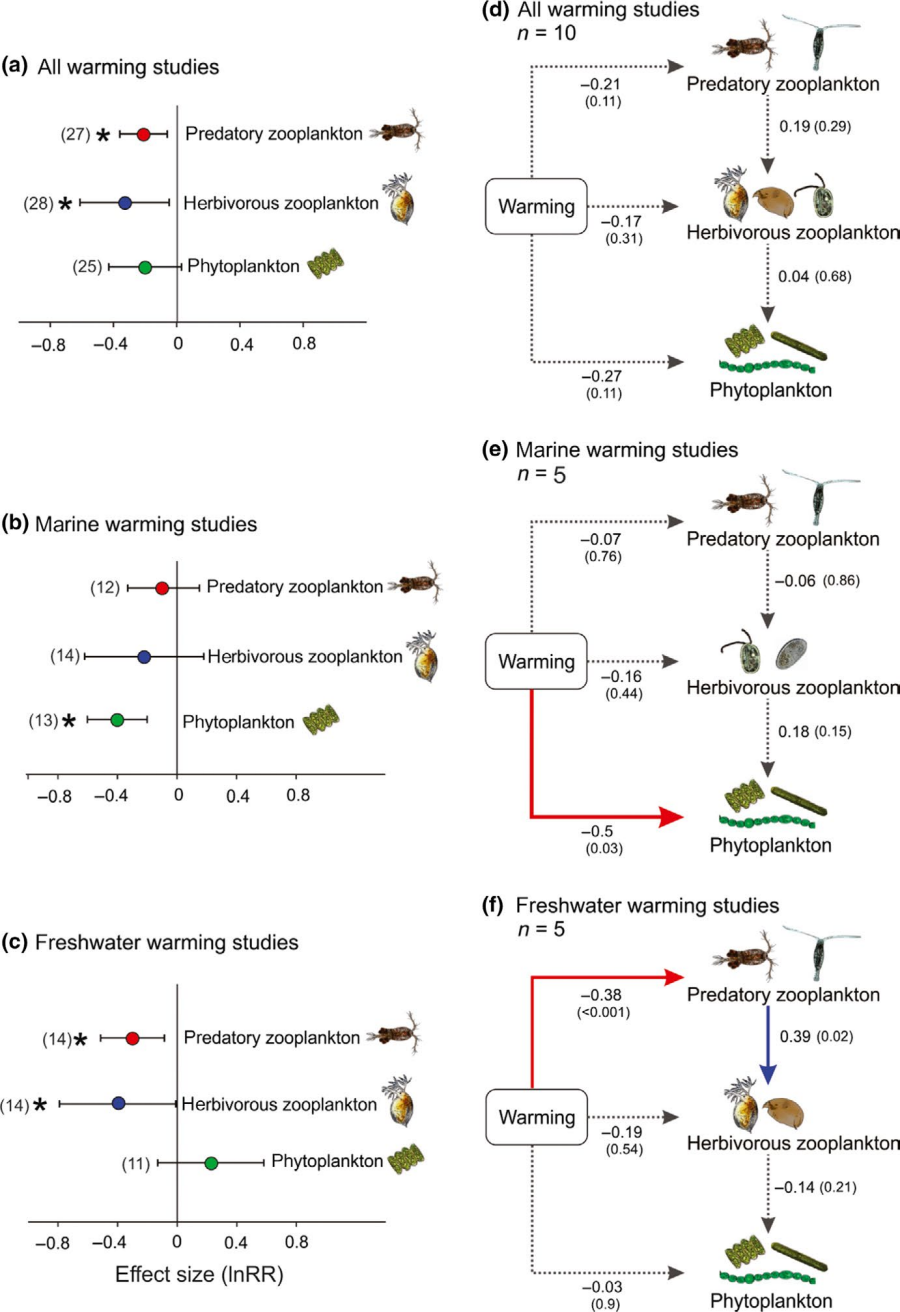
Meta‐analysis of published warming experiments. Shown are response ratios (a, b, c) and SEM path coefficients (d, e, f). Results are displayed for all studies combined or separated by marine and freshwater studies. Path coefficients indicate the strength and direction of the relationship between variables and are shown above arrows with the corresponding *p*‐values displayed in brackets. Bold arrows indicate significant negative (red) and positive (blue) relationships (*p* < .05) and dashed arrows indicate nonsignificant relationships

Across all 28 responses, warming significantly reduced predatory zooplankton by 23% and herbivorous zooplankton by 39%, but did not significantly impact phytoplankton concentration (Figure 4a). A *Q*‐test revealed significant heterogeneity that was reduced by splitting the dataset into marine and freshwater studies. This revealed that warming had a significant negative effect on the concentration of marine phytoplankton (Figure 4b) and a positive, yet not significant, effect on the concentration of freshwater phytoplankton (Figure 4c). In contrast, warming had little effect on marine zooplankton and a significant, negative effect on both trophic levels of freshwater zooplankton (Figure 4b,c).

The meta‐analysis of SEM path coefficients from 10 warming studies with sufficiently detailed data corresponded with these findings, indicating a significant direct effect of warming on phytoplankton in marine ecosystems and a significant direct effect of warming on predatory zooplankton in freshwater ecosystems, but not vice versa (Figure 4e,f). While warming influenced the top and bottom trophic groups in marine and freshwater ecosystems differently, the direct effect of warming on herbivorous zooplankton was comparable. Similar to our experimental results, this effect was weak, not significant, and negative. As in our experiments, the meta‐analysis SEM also revealed weak herbivore–phytoplankton interactions across all studies (Figure 4d) and in both marine and freshwater communities (Figure 4e,f). We also found a stronger predator–herbivore interaction in freshwater compared to marine plankton communities (Figure 4e,f).

## 4 DISCUSSION

Our experiments showed that elevated temperatures primarily impact the top of the plankton food web and suggest that intermediate trophic groups are strongly influenced by altered trophic interactions as opposed to direct effects of warming. Higher trophic levels are generally more susceptible to elevated temperature, as the metabolic demands of consumers are more sensitive to warming than those of primary producers (Lopez‐Urrutia, San Martin, Harris, & Irigoien, 2006). This leads to higher grazing rates and eventually decreased consumer fitness when energy intake by consumers cannot keep up with their metabolic demands (Rall, Vucic‐pestic, Ehnes, Emmerson, & Brose, 2010). Strengthened top‐down control in plankton communities has been demonstrated in other warming experiments (Kratina et al., 2012; O'Connor et al., 2009; Sommer & Lewandowska, 2011), and the top‐down effects we observed with both warming and direct predator removal emphasize that loss of top‐predators can cascade to lower trophic levels.

Structural equation models further supported strong direct effects of warming on top predators, but revealed no direct impacts of warming on two intermediate trophic groups. Instead, the changes in density of mesopredators and herbivores that we observed in our experiments appear to be driven by top‐down control of predators on their prey. In the absence of the abiotic stresses of warming and acidification, experimental reduction of predators and phytoplankton led to markedly similar path coefficients, providing further evidence that trophic interactions mediated by top‐predator declines are a dominant factor controlling intermediate trophic groups. Warming has been shown to alter interactions between trophic groups (O'Connor et al., 2009; Van der Putten, Macel, & Visser, 2010; Yvon‐durocher et al., 2010) and increase the strength of indirect trophic interactions (Barton & Schmitz, 2009; Marquis, Toro, & Pelini, 2014). Intermediate trophic groups are particularly vulnerable to altered species interactions through both direct consumption from predators and nonconsumptive interactions, such as the effects of predation risk, which are reported to increase with warming (Miller, Matassa, & Trussell, 2014).

The negligible direct effect of warming on mesopredators and herbivores in the SEM (Table S1, Figure 3a) suggests that the significant net effects of warming observed in the ANOVA (Table 1, Figure 1) were driven by cascading interactions between the prey species and their predators. This demonstrates the importance of considering how trophic interactions may be altered with climate change. These results also emphasize the importance of partitioning net effects into direct and indirect effects (Alsterberg et al., 2013; Antiquiera et al., 2018). In our analyses, we used ANOVA to estimate the net effects of drivers on each trophic group and SEM to partition these into direct and indirect effects. Doing so provided insights which would not have been revealed by the net effects alone. First, SEM revealed that the significant positive effect of warming on mesopredator density and significant negative effect of warming on herbivore density resulting from the ANOVA were mediated by indirect top‐down trophic interactions as opposed to direct effects of warming on these trophic groups. Secondly, SEM revealed a similar trend when top predator density decreased through direct removal as opposed to warming. We also observed negative net effects of warming on the omnivore and detritivore trophic groups.

Altered trophic interactions led to a major shift in our experimental plankton communities, which was partially driven by a significant increase in mesopredator density with warming and predator removal. Mesopredator release has been described across a range of aquatic and terrestrial ecosystems (Baum & Worm, 2009; Ripple et al., 2014). Our observation of this phenomenon with both warming and direct predator removal in our experimental plankton communities supports the importance of cascading trophic effects. We found that the combined stress of warming and acidification on plankton communities led to differential vulnerability among trophic groups. Some trophic groups were resistant to the interactive effects of warming and acidification, particularly predatory and omnivorous zooplankton, while lower trophic level zooplankton, particularly herbivores and detritivores, were more vulnerable. Furthermore, acidification strengthened the negative effect of warming on herbivore density, while dampening the negative effect of warming on detritivore density. These results show that the combined stresses associated with climate change have the ability to amplify or dampen the singular effects, and this is partly dependent on trophic role.

The interaction strengths from our two experiments were mostly similar; however, a difference in the strength of the herbivore–phytoplankton interaction suggests that warming and acidification weaken herbivore control on phytoplankton. Direct predator removal led to a strong negative herbivore–phytoplankton interaction, but with warming and acidification this interaction was weaker and not significant (Figure 3). Our results also show that acidification directly impacted herbivorous zooplankton density through increased mortality. A physiological impact of either warming or acidification on grazing ability of zooplankton or a reduction in phytoplankton edibility may be driving a weakened herbivore–phytoplankton interaction. While our study was not designed to quantify zooplankton grazing rates or phytoplankton nutritional content, previous studies have noted little impact of acidification on grazing rates, and instead point to decreased algal nutritional quality as the driving force behind altered herbivore–producer interactions (Duarte et al., 2016; Poore et al., 2013).

Altered phytoplankton taxonomic composition can also play a role in changing the strength of zooplankton–phytoplankton interactions. Warming and acidification resulted in a shift from less edible (cyanobacteria) to more edible (diatoms) phytoplankton taxa in our experimental communities (Table 1). The absence of a strong trophic interaction between zooplankton and phytoplankton with this switch from inedible to edible taxa suggests that warming and acidification may have affected the edibility of diatoms. Numerous studies have reported a decrease in the size of marine phytoplankton with increasing temperature (Sommer et al., 2016). With phytoplankton size being an important factor in determining trophic connections (Boyce, Frank, & Leggett, 2015), it is possible that warming reduced interaction strength between zooplankton and phytoplankton by selecting for small cell sizes that are less efficiently grazed by zooplankton.

Our meta‐analysis provides more general evidence across a wide range of plankton communities that warming will exert a stronger direct influence on the top and bottom of food webs. In contrast to the SEM from our experimental data (Figure 3), we did not find strong trophic interactions in any of the meta‐analysis SEMs, apart from a strong predator–herbivore interaction in freshwater plankton communities (Figure 4f). While these results do not support our experimental finding that intermediate trophic groups will be more impacted by changing trophic interactions, they do confirm that warming will have stronger direct effects on the top of the food web than on intermediate trophic groups. It is also important to note that the models in the meta‐analysis differ from those in the experiments as they do not include a mesopredator trophic group.

Our meta‐analysis results also highlight interesting differences between marine and freshwater plankton studies. We detected a stronger predator–herbivore interaction in freshwater compared to marine plankton communities. Contrary to what would be expected for a predator–prey relationship, this interaction was positive. This disparity may be explained by a difference in the zooplankton taxa reported. All freshwater studies (except our own) reported the abundances of copepods and cladocerans, while marine studies reported the abundances of copepods and microzooplankton (ciliates and heterotrophic nanoflagellates). The copepods reported in the freshwater studies are omnivorous and occupy a higher trophic level than the cladocerans; however, it is possible that there was not a strong predator–prey relationship between the two. Interactions between copepods and cladocerans with other species that were present in the experimental communities, but whose abundances were not reported, may have resulted in facilitative interactions, via the copepods improving cladoceran resource availability by preying on competing herbivorous zooplankton. In contrast, microzooplankton comprise a large portion of the copepod diet in the marine experiments, which resulted in the negative, albeit weak, predator–herbivore interaction in marine studies.

In conclusion, our experiments and meta‐analyses show that warming has direct impacts on the top of plankton food webs and that top‐down effects are stronger in shaping the plankton community through cascading interactions. Our results reveal differences in how trophic groups respond to climate change stressors and provide evidence that intermediate trophic groups are more impacted by cascading trophic interactions than by the direct effects of warming and acidification. We show that alterations in interaction chains from the indirect effects of warming, acidification, and predator decline can be equally important as direct effects in restructuring plankton communities under climate change. Accurately forecasting the effects of climate change is not possible without understanding its effects on trophic interactions. We emphasize the need for multitrophic studies of natural plankton communities that partition the net effects of climate change stressors into direct and indirect effects to fully understand the consequences that present and future global change will have on aquatic ecosystems.

## CONFLICT OF INTEREST

None declared.

## AUTHOR CONTRIBUTIONS

G.M. and T.R. jointly designed the study, G.M. conducted the study and analyzed the data, and G.M. and B.W. interpreted the findings and wrote the manuscript.

## Data Availability

The data supporting this study are available at https://doi.org/10.5061/dryad.k98sf7m3c
