# Supplementary figures and images for: Cascading effects of climate change on plankton community structure
Source: Ecol Evol. 2020 Feb 5;10(4):2170–81. doi: 10.1002/ece3.6055 (PMC7042755; doi:10.1002/ece3.6055)

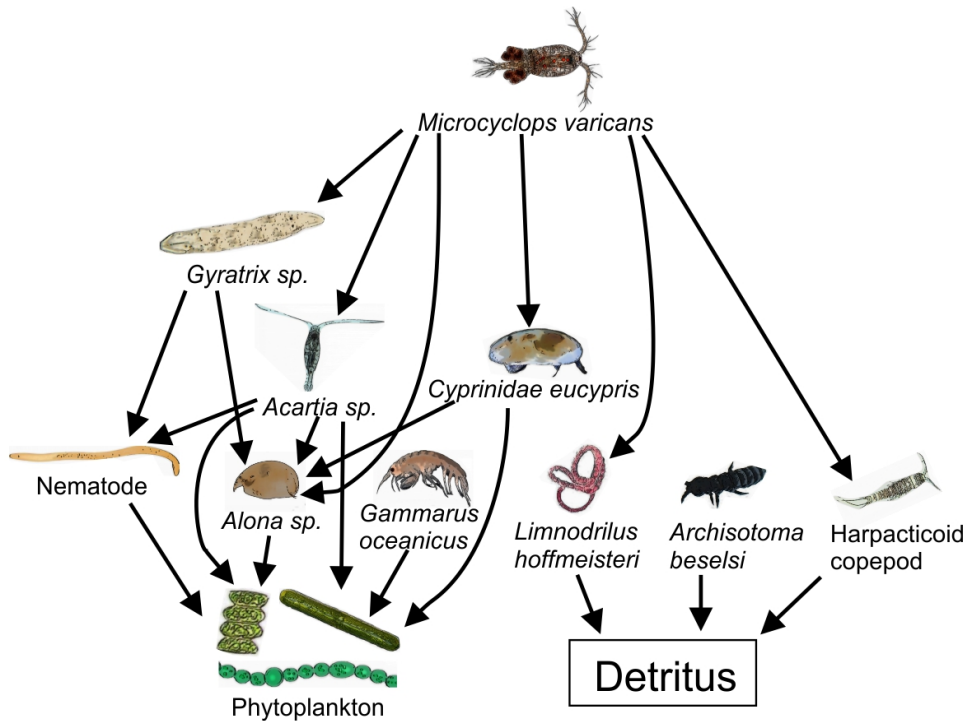

Supplement: Supplementary file 1 [file ECE3-10-2170-s001.pdf]

# A.

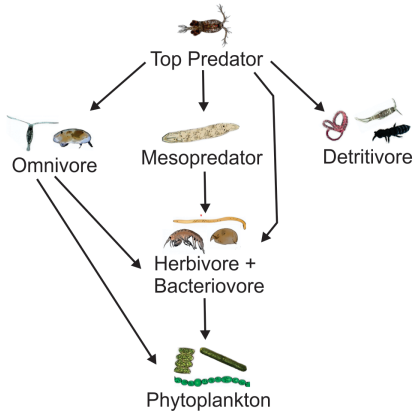

# B.

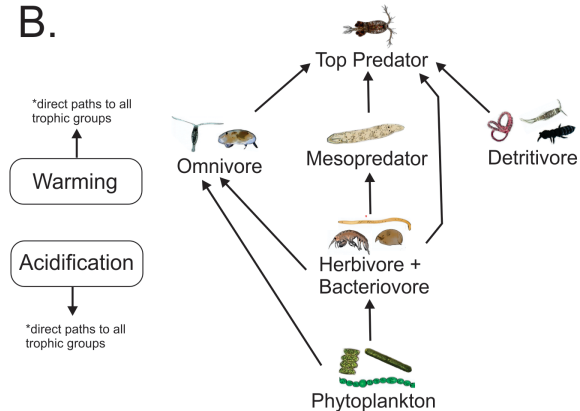

Supplement: Supplementary file 2 [file ECE3-10-2170-s002.pdf]

A.

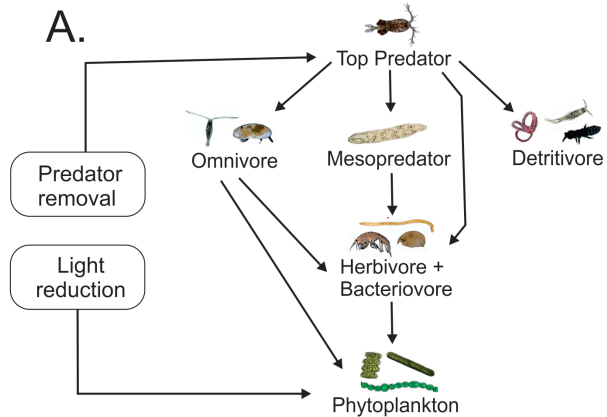

B.

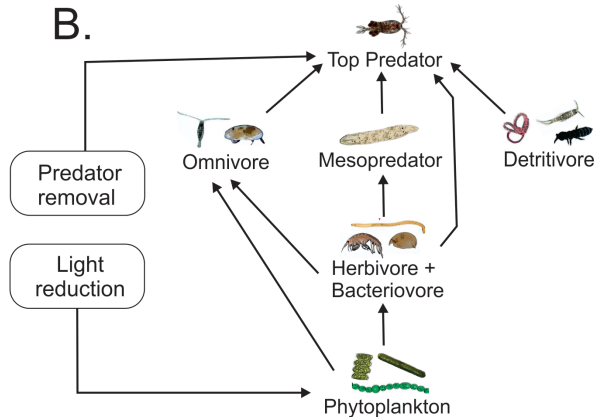

Supplement: Supplementary file 3 [file ECE3-10-2170-s003.pdf]

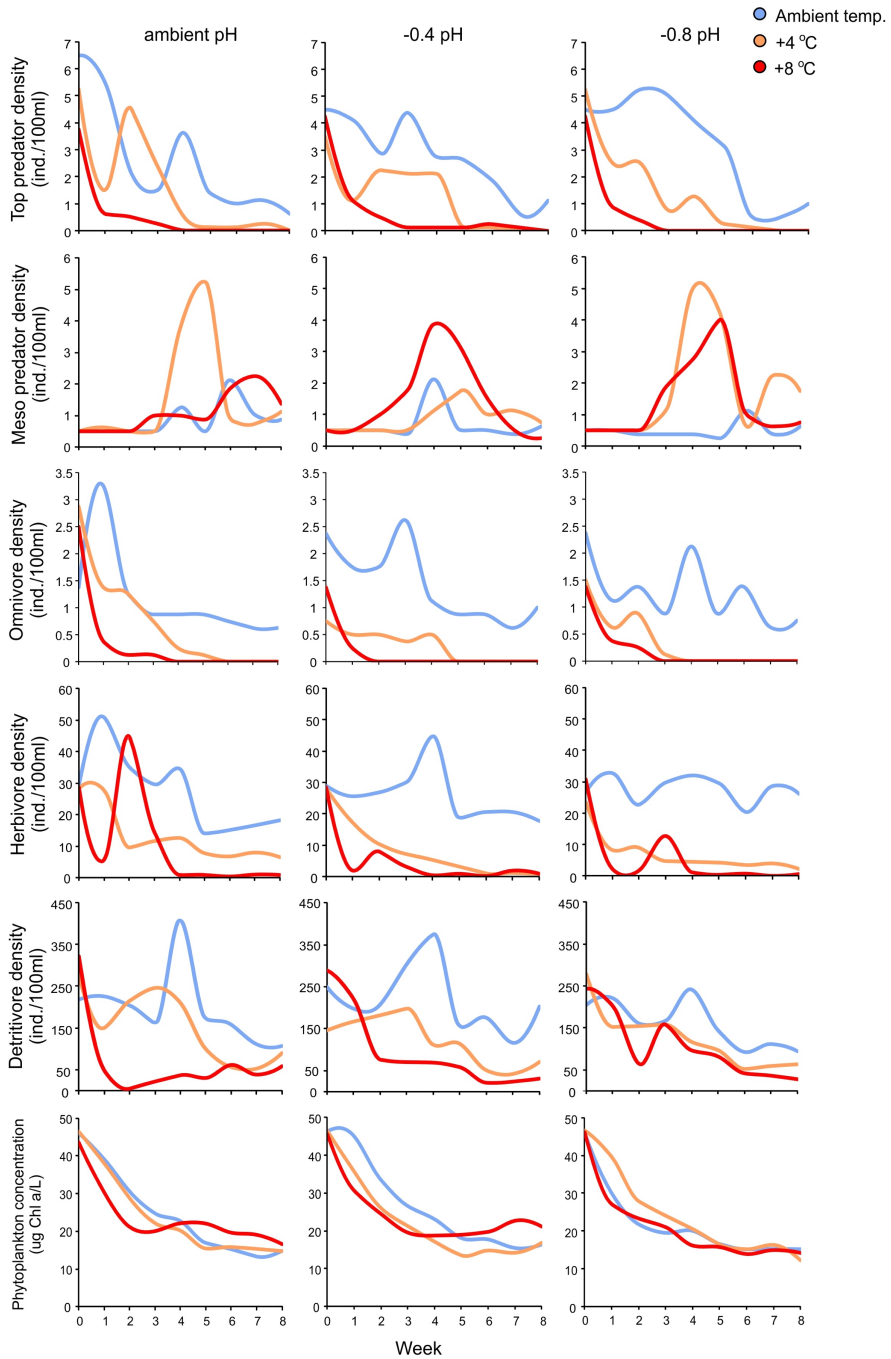

Supplement: Supplementary file 4 [file ECE3-10-2170-s004.pdf]

● No removal  
 ● 50% removal  
 ● 100% removal

12hr light

6hr light

3hr light

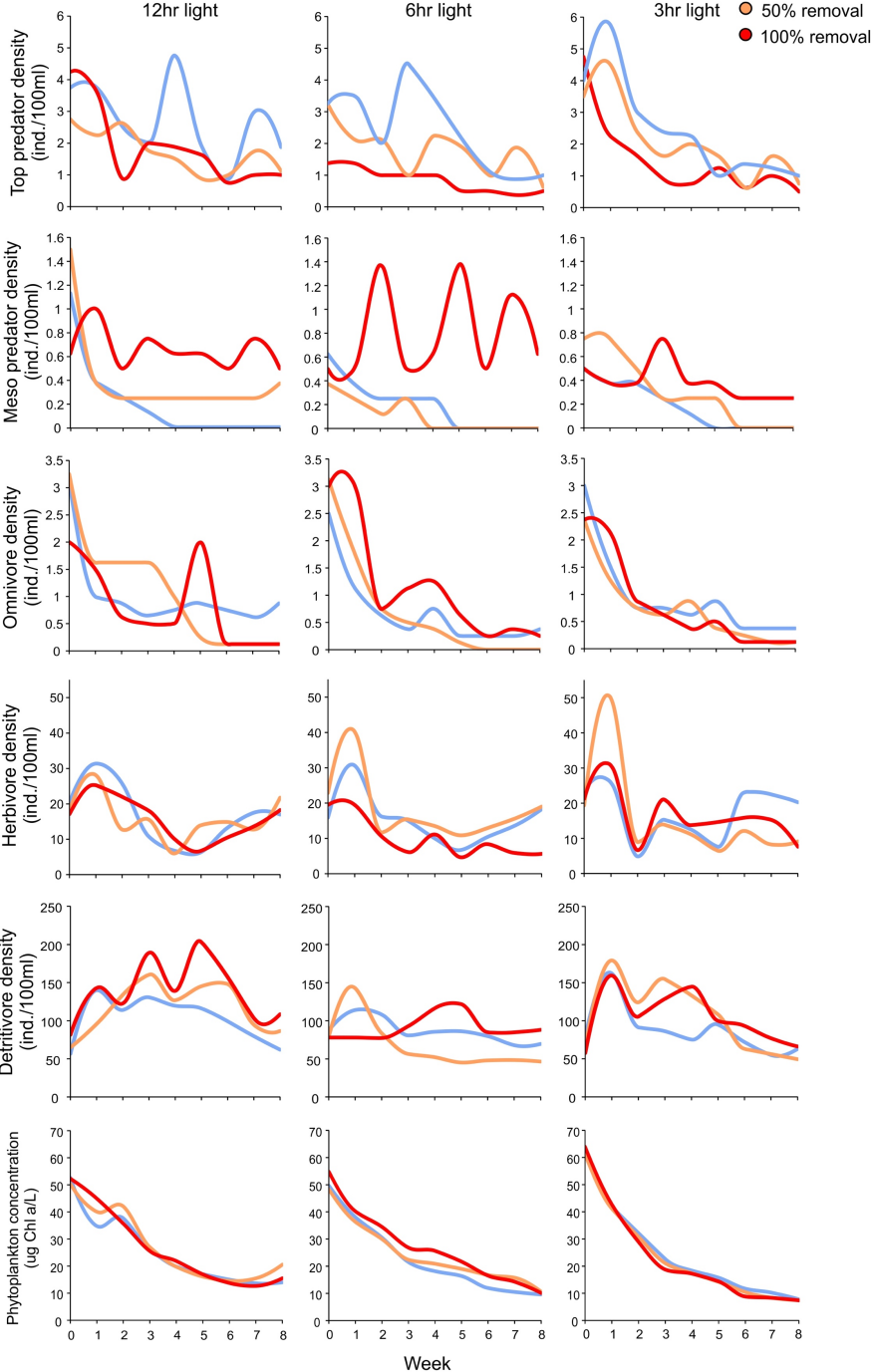

Supplement: Supplementary file 5 [file ECE3-10-2170-s005.pdf]

**(a) all warming studies**  
n = 12

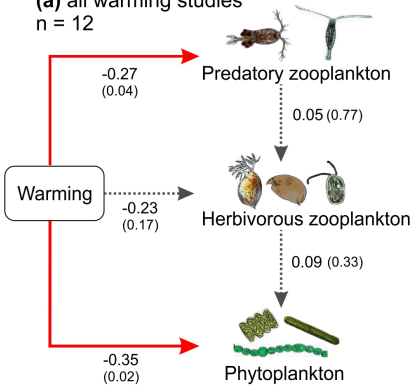

**(b) marine warming studies**  
n = 7

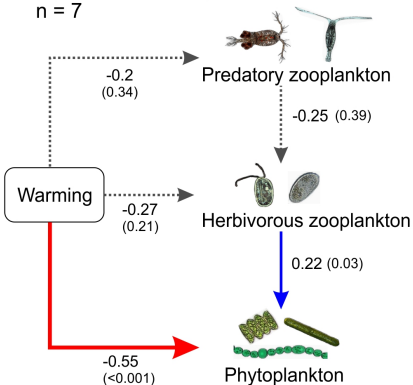

**(c) freshwater warming studies**  
n = 5

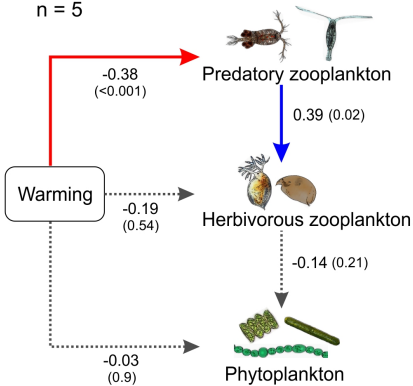

Supplement: Supplementary file 6 [file ECE3-10-2170-s006.pdf]
