# Supplementary material for: Cascading effects of climate change on plankton community structure
Source: Ecol Evol. 2020 Feb 5;10(4):2170–81. doi: 10.1002/ece3.6055 (PMC7042755; doi:10.1002/ece3.6055)
Supplement: Supplementary file 7 [file ECE3-10-2170-s007.docx]

**Appendix S1**

Additional methods:

*Experiment A: warming x acidification*

In experiment A, we elevated the microcosm water temperature by 0, 4, or 8 °C and reduced the pH by 0, 0.4, or 0.8 pH units. Water temperature was manipulated by placing microcosms on heating pads. Averaged across the eight weeks, the temperature in the control microcosms was 21.54 °C (min. = 20.94 °C, max. = 22.06 °C). The moderate warming treatment increased average temperature by 4 °C to 25.55 °C (min. = 24.81 °C, max. = 26.44 °C), and the severe warming treatment increased average temperature by 7.7 °C to 29.22 °C (min. = 28.11 °C, max. = 29.75 °C). These temperatures are well within the range of summertime *in situ* temperatures found in rockpools at our sampling site. Sulfuric acid (H_2_SO_4_) was used to reduce the pH of the microcosms. Each week the pH was measured prior to acid additions, and acid was added to reduce the ambient pH by either 0.4 or 0.8 units. Target pH was determined according to the ambient pH of the individual microcosms before the start of the experiment, which ranged from 7.57 to 8.09. The pH was continually monitored and acid was added on a weekly basis, as needed to continually reduce the pH to the prescribed treatment levels. Averaged across the eight weeks, the pH in the control microcosms was 8.08 (min. = 7.97, max. = 8.15). The moderate acidification treatment decreased average pH measured after weekly acid addition by 0.59 to 7.49 (min. = 7.16, max. = 7.67), and the severe acidification treatment decreased average pH measured after acid addition by 0.84 to 7.24 (min. = 7.02, max. = 7.41).

*Experiment B: top predator removal x light reduction*

Experiment B consisted of three predator removal treatments: no removal, 50% removal, and 100% removal, crossed with three light reduction treatments: 12 hours of light per day, 6 hours of light per day, and 3 hours of light per day. We performed weekly predator removals by examining the entire contents of each microcosm under a stereomicroscope and manually removing 0%, 50%, or 100% of the total number of cyclopoid copepods present. Only adult copepods were removed since juvenile and nauplii stages could not easily be distinguished from other copepod taxa. The removal of cyclopoid copepods resulted in a significant gradient in top predator abundance among the three removal treatments (p < 0.001). Averaged over the course of the experiment, the abundance of cyclopoid copepods in the control treatment (30.04 +- 0.89 SE) was 30% higher than the 50% removal treatment (20.89 +- 1.95 SE, p = 0.005) and 67% higher than the 100% removal treatment (9.83 +- 1.5 SE, p < 0.001). The light reduction treatments resulted in a significant decrease in total phytoplankton concentration (p = 0.04) and a reorganization of the phytoplankton community (p < 0.001), with lower concentrations of cyanobacteria and cryptophyte phytoplankton (p < 0.001), higher concentration of green algae (p = 0.024), and no change in diatoms (p = 0.493).

**Table S1**. Significant (p < 0.05) standardized path coefficients obtained from SEM for direct, indirect, and total effects of warming and acidification on trophic group densities. Indirect effects are calculated as the product of direct effects and total effects are calculated as the sum of direct and indirect effects. Corresponding path diagram is shown in Figure 3a.

|  | Warming | | | Acidification | | |
| --- | --- | --- | --- | --- | --- | --- |
|  | Direct effect | Indirect effect | Total effect | Direct effect | Indirect effect | Total effect |
| Top predator | -0.93 | 0 | -0.93 | 0 | 0 | 0 |
| Mesopredator  Omnivore | 0  -0.88 | +0.46  0 | +0.46  -0.88 | 0  0 | 0  0 | 0  0 |
| Herbivore | 0 | -0.81 | -0.81 | -0.3 | 0 | -0.3 |
| Phytoplankton  Detritivore | 0  -0.8 | 0  0 | 0  -0.8 | 0  0 | 0  0 | 0  0 |

**Table S2**. Significant (p < 0.05) standardized path coefficients obtained from SEM for direct, indirect, and total effects of top predator removal and light reduction on trophic group densities. Indirect effects are calculated as the product of direct effects and total effects are calculated as the sum of direct and indirect effects. Corresponding path diagram shown in Figure 3b.

|  | Predator removal | | | Light reduction | | |
| --- | --- | --- | --- | --- | --- | --- |
|  | Direct effect | Indirect effect | Total effect | Direct effect | Indirect effect | Total effect |
| Top predator | -0.73 | 0 | -0.73 | 0 | 0 | 0 |
| Mesopredator | 0 | +0.38 | +0.38 | 0 | 0 | 0 |
| Herbivore | 0 | -0.20 | -0.20 | 0 | 0 | 0 |
| Phytoplankton | 0 | +0.08 | +0.08 | -0.45 | 0 | -0.45 |

**Table S3.** Information on the 16 studies (28 responses) included in meta-analysis.

| Study | Reference | Temp. increase (°C) | Lat. | Long. | Habitat | Experiment length (days) | Included in SEM meta-analysis? |
| --- | --- | --- | --- | --- | --- | --- | --- |
| 1 | Aberle et al. 2012 | 6 | 54.433 | 10.243 | Marine | 98 | yes |
| 2 | Aberle et al. 2015 | 6 | 54.433 | 10.243 | Marine | 38 | yes |
| 3 | Graham and Vinebrooke 2009 | 0.7 | 49.670 | -93.750 | Freshwater | 50 | no |
| 4 | Horn et al. 2016 | 6 | 54.433 | 10.243 | Marine | 21 | yes |
| 5 | Lengfellner 2008 | 2 | 54.433 | 10.243 | Marine | 70 | yes |
| 5 | Lengfellner 2008 | 2 | 54.433 | 10.243 | Marine | 80 | yes |
| 5 | Lengfellner 2008 | 4 | 54.433 | 10.243 | Marine | 70 | yes |
| 5 | Lengfellner 2008 | 4 | 54.433 | 10.243 | Marine | 80 | yes |
| 5 | Lengfellner 2008 | 6 | 54.433 | 10.243 | Marine | 70 | yes |
| 5 | Lengfellner 2008 | 6 | 54.433 | 10.243 | Marine | 80 | yes |
| 6 | Lewandowska et al. 2014 | 6 | 54.433 | 10.243 | Marine | 35 | yes |
| 7 | Murphy et al. (this study) | 4 | 43.491 | -65.791 | Freshwater | 56 | yes |
| 7 | Murphy et al. (this study) | 8 | 43.491 | -65.791 | Freshwater | 56 | yes |
| 8 | Nicolle et al. 2012 | 3 | 52.7 | 13.2 | Freshwater | 83 | no |
| 9 | Ozen et al. 2013 | 2.7 | 56.242 | 9.546 | Freshwater | 120 | no |
| 10 | Sommer et al. 2007 | 2 | 54.433 | 10.243 | Marine | 90 | yes |
| 10 | Sommer et al. 2007 | 4 | 54.433 | 10.243 | Marine | 90 | yes |
| 10 | Sommer et al. 2007 | 6 | 54.433 | 10.243 | Marine | 90 | yes |
| 11 | Sorf et al. 2015 | 2.7 | 56.242 | 9.546 | Freshwater | 150 | no |
| 11 | Sorf et al. 2015 | 4.1 | 56.242 | 9.546 | Freshwater | 150 | no |
| 12 | Strecker et al. 2004 | 3.6 | 51.178 | -115.57 | Freshwater | 50 | no |
| 12 | Strecker et al. 2004 | 3.6 | 51.178 | -115.57 | Freshwater | 50 | no |
| 12 | Strecker et al. 2004 | 3.6 | 51.178 | -115.57 | Freshwater | 50 | no |
| 13 | Thompson et al. 2008 | 6 | 51.62 | -115.85 | Freshwater | 36 | yes |
| 13 | Thompson et al. 2008 | 6 | 51.62 | -115.85 | Freshwater | 36 | yes |
| 14 | Vidussi et al. 2011 | 3 | 43.4 | 3.68 | Marine | 10 | no |
| 15 | Yvon-Durocher et al. 2011 | 4 | 50.22 | -2.16 | Freshwater | 150 | yes |
| 16 | Yvon-Durocher et al. 2015 | 4 | 50.22 | -2.16 | Freshwater | 1825 | yes |

**Table S4**. Experiment A model selection for top-down structural equation model. We used corrected Akaike information criterion (AICc) for model selection. We evaluated model fit using the Fisher’s C statistic. Variable abbreviations: TEMP = temperature, pH = pH, TP = top predator, MP = mesopredator, OM= omnivore, DT = detritivore, HB = herbivore + bacteriovore, PP = phytoplankton.

| Model selection steps | Pathways removed from full model (in addition to pathways removed from previous steps) | AICc | ΔAICc | Fisher’s C | p-value |
| --- | --- | --- | --- | --- | --- |
| Full model | - | 1084.38 | 958.46 | 26.37 | 0.023 |
| 1 | TEMP 🡪 MP | 795.47 | 669.55 | 26.39 | 0.049 |
| 2 | OM 🡪 HB | 621.97 | 496.05 | 26.39 | 0.091 |
| 3 | pH 🡪 MP | 508.50 | 382.58 | 26.75 | 0.142 |
| 4 | OM 🡪 PP | 426.08 | 300.16 | 26.85 | 0.217 |
| 5  6  7  8  9  10  11  12  Final | TP 🡪 DT  pH 🡪 TP  pH 🡪 DT  HB 🡪 PP  TP 🡪 OM  pH 🡪 PP  TEMP 🡪 PP  pH 🡪 OM  TEMP 🡪 HB | 371.20  325.04  289.09  259.32  243.18  231.49  134.84  128.72  125.92 | 245.28  199.12  163.17  133.4  117.26  105.57  8.92  2.8  0 | 28.49  29.26  30.30  31.24  35.06  39.59  21.93  24.78  28.96 | 0.240  0.299  0.349  0.404  0.325  0.234  0.464  0.418  0.313 |
|  |  |  |  |  |  |

**Table S5**. Experiment A model selection for bottom-up structural equation model. We used corrected Akaike information criterion (AICc) for model selection. We evaluated model fit using the Fisher’s C statistic. Variable abbreviations: TEMP = temperature, pH = pH, TP = top predator, MP = mesopredator, OM= omnivore, DT = detritivore, HB = herbivore + bacteriovore, PP = phytoplankton.

| Model selection steps | Pathways removed from full model (in addition to pathways removed from previous steps) | AICc | ΔAICc | Fisher’s C | p-value |
| --- | --- | --- | --- | --- | --- |
| Full model | - | 797.98 | 666.48 | 26.66 | 0.045 |
| 1 | MP 🡪 TP | 624.92 | 493.42 | 26.79 | 0.083 |
| 2 | PP 🡪 OM | 513.20 | 381.7 | 27.53 | 0.121 |
| 3 | pH 🡪 DT | 434.41 | 302.91 | 28.47 | 0.161 |
| 4 | OM 🡪 TP | 378.16 | 246.66 | 30.04 | 0.184 |
| 5  6  7  8  9  10  11  Final | TEMP 🡪 MP  DT 🡪 TP  pH 🡪 MP  pH 🡪 PP  TEMP 🡪 PP  pH 🡪 OM  pH 🡪 TP  HB 🡪 TP | 336.24  309.63  282.16  260.38  183.98  172.09  134.12  131.50 | 204.74  178.13  150.66  128.88  52.48  40.59  2.62  0 | 32.06  36.01  38.21  40.79  36.66  38.48  33.06  37.404 | 0.191  0.142  0.144  0.137  0.187  0.199  0.160  0.110 |
|  |  |  |  |  |  |

**Table S6**. Experiment B model selection for top-down structural equation model. We used corrected Akaike information criterion (AICc) for model selection. We evaluated model fit using the Fisher’s C statistic. Variable abbreviations: TP = top predator, MP = mesopredator, OM= omnivore, DT = detritivore, HB = herbivore + bacteriovore, PP = phytoplankton.

| Model selection steps | Pathways removed from full model (in addition to pathways removed from previous steps) | AICc | ΔAICc | Fisher’s C | p-value |
| --- | --- | --- | --- | --- | --- |
| Full model | - | 244.77 | 21.26 | 44.39 | 0.11 |
| 1 | + TP 🡪 HB | 223.51 | 57.73 | 44.54 | 0.16 |
| 2 | + TP 🡪 OM | 165.78 | 11.29 | 42.29 | 0.16 |
| 3 | + OM 🡪 PP | 154.49 | 70.58 | 43.35 | 0.19 |
| Final | + TP 🡪 DT | 83.91 | 0 | 43.25 | 0.19 |
|  |  |  |  |  |  |
|  |  |  |  |  |  |

**Table S7**. Experiment B model selection for bottom-up structural equation model. We used corrected Akaike information criterion (AICc) for model selection. We evaluated model fit using the Fisher’s C statistic. Variable abbreviations: TP = top predator, MP = mesopredator, OM= omnivore, DT = detritivore, HB = herbivore + bacteriovore, PP = phytoplankton.

| Model selection steps | Pathways removed from full model (in addition to pathways removed from previous steps) | AICc | ΔAICc | Fisher’s C | p-value |
| --- | --- | --- | --- | --- | --- |
| Full model | - | 195.22 | 15.15 | 48.76 | 0.029 |
| 1 | HB 🡪 TP | 180.07 | 33.55 | 49.03 | 0.046 |
| 2 | + DT 🡪 TP | 146.52 | 9.66 | 39.26 | 0.046 |
| 3 | + OM 🡪 TP | 136.86 | 7.24 | 40.23 | 0.063 |
| 4 | + PP 🡪 OM | 129.62 | 40.56 | 42.01 | 0.071 |
| Final | + MP 🡪 TP | 89.06 | 0 | 32.9 | 0.03 |
|  |  |  |  |  |  |

**Table S8**. Summary statistics for Experiment A final structural equation model. Trophic group densities are log(x+1)-transformed. Temperature and pH are square root-transformed. Standardized estimates are scaled by standard deviations. Corresponding path diagram is shown in Figure 3a. Note that the pathway from Acidification to Herbivore in Fig. 3a is represented as a negative relationship to account for the fact that acidification refers to decreased pH.

| Response | Predictor | Estimate | SE | DF | p-value | Std. Estimate |
| --- | --- | --- | --- | --- | --- | --- |
| Top Predator | Temperature | -0.61 | 0.04 | 34 | <0.001 | -0.93 |
| Mesopredator  Herbivore  Herbivore  Herbivore  Detritivore  Omnivore | Top Predator  Mesopredator  Top Predator  pH  Temperature  Temperature | -0.31  -0.72  1.49  1.86  -0.62  -0.40 | 0.09  0.27  0.18  0.48  0.08  0.04 | 34  32  32  32  34  34 | 0.003  0.013  <0.001  <0.001  <0.001  <0.001 | -0.49  -0.23  0.76  0.30  -0.80  -0.88 |

**Table S9**. Summary statistics for Experiment B final structural equation model. Trophic group densities are log(x+1)-transformed. Standardized estimates are scaled by standard deviations. Corresponding path diagram is shown in Figure 3b.

| Response | Predictor | Estimate | SE | DF | p-value | Std. Estimate |
| --- | --- | --- | --- | --- | --- | --- |
| Top Predator  Mesopredator | Removal treatment  Top Predator | -0.09  -0.51 | 0.01  0.14 | 34  34 | <0.001  0.001 | -0.73  -0.52 |
| Herbivore  Herbivore  Phytoplankton  Phytoplankton | Mesopredator  Omnivore  Herbivore  Light treatment | -0.99  -0.58  -0.17  -0.042 | 0.25  0.27  0.06  0.01 | 33  33  33  33 | <0.001  0.037  0.006  0.002 | -0.54  -0.29  -0.41  -0.45 |
|  |  |  |  |  |  |  |

**Table S10**. Summary statistics for meta-analysis (Fig 4 a,b,c).

|  | Trophic group | Effect size (lnRR) | n | 95% CI | Q-stat |
| --- | --- | --- | --- | --- | --- |
| All studies (Fig. 4a) | Predator  Herbivore  Phytoplankton | -0.21  -0.33  -0.20 | 27  28  25 | 0.150  0.282  0.230 | 280  268  2750 |
| Marine (Fig. 4b)  Freshwater (Fig. 4c) | Predator  Herbivore  Phytoplankton  Predator  Herbivore  Phytoplankton | -0.09  -0.22  -0.40  -0.30  -0.40  0.23 | 13  14  12  14  14  11 | 0.241  0.400  0.201  0.214  0.390  0.356 | 40.93  25.71  77.09  238.36  270  272.81 |

**Figure legends**

**Figure S1.** Food web in experimental rock pool community. Arrows represent known feeding interactions.

**Figure S2.** Full *a priori* model for Experiment A describing the hypothesized causal mechanisms for how warming and acidification, and subsequent biotic interactions, interact to alter plankton trophic composition. The first model (A) includes top-down controls on the plankton community and the second model (B) includes bottom-up controls on the plankton community. Pathways between trophic groups are based on known feeding interactions.

**Figure S3.** Full *a priori* model for Experiment B describing the hypothesized causal mechanisms for how top predator removal and light reduction, and subsequent biotic interactions, interact to alter plankton trophic composition. The first model (A) includes top-down controls on the plankton community and the second model (B) includes bottom-up controls on the plankton community. Pathways between trophic groups are based on known feeding interactions.

**Figure S4.** Time trends of zooplankton trophic group density and total phytoplankton concentration in warming and acidification treatments (Experiment A). Interpolated time series for the average densities of each trophic group and average phytoplankton concentration (n = 4). Interpolation of each time series was performed using the Akima method.

**Figure S5.** Time trends of zooplankton trophic group density and total phytoplankton concentration in top predator removal and light reduction treatments. Interpolated time series for the average densities of each trophic group and average phytoplankton concentration (n = 4). Interpolation of each time series was performed using the Akima method.

**Figure S6.** Structural equation model meta-analysis results including studies that significantly differ from *a priori* model. The path coefficients, which indicate the strength and direction of the relationship between variables, are shown above arrows with the corresponding p-values displayed in brackets. Bold arrows indicate significant negative (red) and positive (blue) relationships (p < 0.05) and dashed arrows indicate non-significant relationships.
